# Does Preoperative Mixed‐Growth Urine Culture Increase the Risk of Urinary Tract Infection After Ureteroscopic Stone Treatment? A Retrospective Study

**DOI:** 10.1002/hsr2.73064

**Published:** 2026-08-26

**Authors:** Khalid Elsayed Mahmoud, Ahmad R. Al‐Qudimat, Ahmed Khaled Alhusban, Raed M. Al‐Zoubi, Mai Elaarag, Abdulkader Alobiedly, Ibrahim A. khalil, Abdulla AlAnsari

**Affiliations:** ^1^ Urology Division, Department of Surgery Hamad Medical Corporation Doha Qatar; ^2^ Surgical Research Section, Department of Surgery Hamad Medical Corporation Doha Qatar; ^3^ Department of Biomedical Sciences, QU‐Health, College of Health Sciences Qatar University Doha Qatar; ^4^ Department of Chemistry Jordan University of Science and Technology Irbid Jordan

**Keywords:** contamination, kidney stones, postoperative complications, ureteroscopy, urinary tract infections, urine culture

## Abstract

**Background:**

Ureteroscopy (URS) has emerged as a leading treatment option for urinary stones, with its usage growing from 30% to 70% over the last 20 years. Post‐URS complications, particularly infectious issues like UTIs, remain a concern, occurring in up to 25% of cases. This study aims to explore the relationship between preoperative mixed‐growth urine culture and postoperative outcomes of URS, addressing a critical gap in understanding the value and influence of these cultures.

**Methodology:**

This retrospective study included adult patients (18–65 years) who underwent RIRS or R‐URS for ureteral or renal stones at a tertiary care center between January 1, 2020, and May 1, 2024. Patients were categorized by preoperative urine culture (no growth vs. mixed growth), and consecutive cases were included until equal group sizes were achieved. Univariable and multivariable logistic regression were used to identify factors associated with mixed growth and to evaluate outcomes related to mixed growth.

**Results:**

The cohort included two equal‐sized groups (*n* = 73 each) based on urine culture results. The cohort comprised 52.05% males and 47.95% females. The mixed growth group exhibited significantly lower hemoglobin levels (*p* < 0.001) and higher bacterial counts (*p* < 0.001). ICU admission showed a trend toward significance (*p* = 0.08), while other factors like pre‐stented ureters (*p* = 0.09) and type of surgery (*p* = 0.092) did not reach significance. Mixed bacterial growth on urine culture was associated with significantly higher rates of postoperative UTI (17.8% vs. 2.7%, *p* = 0.003), readmission (17.8% vs. 0%, *p* < 0.001), and fever (15.1% vs. 0%, *p* < 0.001), with a non‐significant increase in ICU admission (4.1% vs. 0%, *p* = 0.08). Male gender was independently associated with a lower likelihood of mixed bacterial growth (OR = 0.25, 95% CI: 0.11–0.52, *p* = 0.001), whereas mixed growth was associated with increased odds of postoperative UTI on univariate analysis.

**Conclusion:**

Mixed bacterial growth on preoperative urine culture is associated with a higher risk of postoperative infectious complications following RIRS and R‐URS. Female patients appear to be at higher risk for mixed growth, highlighting the importance of careful preoperative assessment and targeted perioperative management to optimize outcomes.

## 1 Introduction/Background

Urolithiasis, or kidney stone disease, has become increasingly common in modern societies, driven by factors such as rising obesity rates, diabetes, and dietary changes [1, 2, 3]. Ureteroscopy (URS) has emerged as a leading treatment option for urinary stones, with its usage growing from 30% to 70% over the last 20 years [4, 5]. However, post‐URS complications, particularly infectious issues like UTIs, remain a concern, occurring in up to 25% of cases [6, 7].

Several modalities for urinary stone management exist and include extracorporeal shockwave lithotripsy (SWL), ureteroscopy (URS), and percutaneous nephrolithotomy (PCNL) [4], Compared to SWL or PCNL, the use of URS has increased from 30% to 70% over the past 20 years [8, 9], Following URS, the overall rate of complications varies between 9% and 25% although the majority of these are minor and does not require intervention [10, 11, 12]. Infectious complications ranging from fever, systemic inflammatory response syndrome, to urinary tract infection are some of the more common post‐ureteroscopy complications, alongside hematuria and post‐operative pain [12], with overall complication rates of up to 25% [13, 14].

Urinary tract infections (UTIs) frequently coincide with urolithiasis both as a cause and a complication [15]. Prior to undergoing endoscopic procedures for urolithiasis, patients should undergo a comprehensive preoperative assessment, which includes a detailed medical history and physical examination, as well as essential preoperative blood tests, such as basic metabolic panel (BMP) and a complete blood count (CBC). AUA guidelines recommend obtaining urine cultures only in patients with clinical or laboratory signs of infection [16]. Furthermore, many patients with nephrolithiasis have sterile pyuria due to local inflammation and trauma from the stone. Given these pragmatic challenges with using urinalysis alone, obtaining a urine culture prior to endourologic intervention in all patients is non‐controversial in clinical practice [17]. In our institution, urine cultures were routinely performed preoperatively for all patients undergoing ureteroscopy as part of standard pre‐surgical assessment

Urine culture results are generally reported as no growth, positive growth, or mixed growth. Mixed‐growth urine cultures commonly reflect specimen contamination during urine collection but may also represent polymicrobial bacteriuria. Cultures with positive results for bacteria will typically lead to the appropriate preoperative prescription of an antibiotic; conversely, a negative urine culture usually indicates a non‐increased risk of postoperative infection and typically does not necessitate outpatient preoperative antibiotics. For patients with mixed‐growth urine cultures, a repeat sample may be obtained. The microscopy lab may report contaminated cultures as such, but are also suggested by the presence of epithelial cells on urine microscopy [16].

The number of colony‐forming units per milliliter (CFU/mL) is the standard method for determining whether a UC is positive, but its interpretation may be complicated by contamination [18]. True sample contamination can occur at various points in a culture, such as before sample processing during the collection step from surrounding vaginal, perineal, and epidermal skin flora [18]. However, laboratories report a median of 15% of urine cultures as contaminated for the growth of two or more isolates in quantities greater than or equal to 10^4^ CFU/mL [19].

A significant gap exists in the literature, particularly regarding the presence of contaminants or mixed flora in cultured urine samples. The most recent clinical care guidelines lack specific statements on how to address this issue. Moreover, previous studies have demonstrated that contaminated cultures can impede timely treatment by wasting resources through repeated testing and, most critically, lead to the inappropriate use of antibiotics [19]. Our aim of this study is to explore the effect of mixed‐growth urine cultures on postoperative outcomes after ureteroscopy in our center. This research is expected to be of significant importance, as ureteroscopy is arguably the most common clinical treatment offered to patients with urolithiasis at our facility. Furthermore, urolithiasis is a prevalent urologic condition among residents and locals in Qatar due to various factors, including the hot climate and lifestyle habits, among others. The study's findings should help us formulate national guidelines for managing patients with mixed growth in urine cultures prior to surgical intervention. Additionally, it may allow us to limit unnecessary antibiotic use, which can be both financially demanding and contribute to increased bacterial resistance.

## 2 Materials and Methods

### 2.1 Study Design and Setting

This retrospective study included patients who underwent RIRS and R‐URS at the Ambulatory Care Center between January 1, 2020, and May 1, 2024. Patients were categorized into two groups based on preoperative urine culture results (no growth vs. mixed growth). To enable balanced comparisons between groups and reduce the effect of unequal group size on statistical analysis, consecutive patients were included until group sizes were equal.

### 2.2 Population

The inclusion criteria were adult patients aged 18 to 65 years with stone disease in the ureter and kidney, including those with either multiple or single stones, of all genders, and both elective cases and emergency cases presenting with acute ureteric obstruction requiring urgent intervention. The exclusion criteria were pediatric patients, patients who underwent RIRS or S‐URS for conditions other than stone disease, and patients with a positive urine culture.

### 2.3 Definitions

Mixed‐growth urine culture was defined according to the institutional microbiology laboratory report as the growth of two or more bacterial organisms in a single urine specimen [20]. Mixed growth commonly reflects specimen contamination during urine collection but may also represent polymicrobial bacteriuria. Throughout this study, patients were classified according to the laboratory‐reported urine culture result (no growth or mixed‐growth urine culture).

Emergency cases were defined as patients requiring urgent intervention because of acute ureteric obstruction associated with uncontrolled pain despite medical therapy or acute deterioration in renal function secondary to ureteric obstruction [21].

### 2.4 Procedures

Preoperative urine cultures were obtained within 1–2 weeks prior to elective ureteroscopy in accordance with institutional protocol. According to the AUA guidelines [16]. All patients received routine preoperative antibiotic prophylaxis prior to ureteroscopy in accordance with institutional policy and consistent with guideline recommendations. Room‐temperature normal saline is used as the irrigation fluid through manual irrigation. Semi‐rigid ureteroscopy is employed for ureteric stones, with a size ranging from 7.5 Fr to 9.5 Fr, while single‐use flexible ureteroscopy (Innovex, 9.6 Fr) is used for kidney stones, with or without a ureteric access sheath (Boston Scientific Navigator, 11/13 Fr) as needed. Postoperative antibiotics were administered for 5 days according to institutional protocol.

For emergency cases, urine specimens were obtained before intervention as part of the routine emergency evaluation. However, urine culture results were not immediately available and were typically reported after the procedure. Consequently, surgical decision‐making was based on the patient's clinical presentation, laboratory findings, and imaging at the time of presentation rather than urine culture results.

### 2.5 Ethical Considerations

All research activities were conducted in accordance with the Declaration of Helsinki, Good Clinical Practice (GCP), and the regulatory requirements of the Ministry of Public Health (MOPH) in Qatar. The study protocol was approved by the Institutional Review Board (IRB) of HMC (Approval MRC‐01‐24‐343).

### 2.6 Statistical Analysis

Descriptive statistics were used to summarize patient characteristics, operative details, and postoperative outcomes. Continuous variables were expressed as mean ± standard deviation (SD) for normally distributed data, or as median with interquartile range (IQR) for non‐normally distributed data. Categorical variables were reported as frequencies and percentages. Comparisons between groups (No growth vs. Mixed growth) were performed using the independent *t*‐test for normally distributed continuous variables and the Mann‐Whitney *U* test for non‐normally distributed continuous variables. Chi‐square (χ^2^) or Fisher's exact test was used for categorical variables, as appropriate. To identify factors associated with mixed growth, a univariate logistic regression analysis was conducted to estimate odds ratios (ORs) with 95% confidence intervals (CIs). Variables with a *p*‐value < 0.1 in univariate analysis were included in the multivariable logistic regression model to adjust for potential confounders. The final multivariable model was built using a stepwise backward selection approach, with significance set at *p* < 0.05. All statistical analyses were conducted using Stata version 17, and a *p*‐value < 0.05 was considered statistically significant.

## 3 Results

### 3.1 Patient Characteristics

A total of 146 patients were included, comprising two groups of 73 patients each with no growth and mixed growth on preoperative urine culture, respectively. The proportion of females was significantly higher in the mixed growth group (48/73; 65.75%) compared to the no growth group (*p* < 0.001). Males were more prevalent in the no‐growth group (51/73; 69.86%). The mean BMI was comparable between groups (*p* = 0.62). Regarding ethnicity, the distribution of non‐Arab and Arab patients was similar between the two groups (*p* = 0.41). The prevalence of diabetes mellitus (DM) (*p* = 0.21), hypertension (HTN) (*p* = 0.83), anemia (*p* = 0.15), and dyslipidemia (*p* = 0.19) did not significantly differ between groups. Preoperative laboratory values did not show significant differences in WBC count (*p* = 0.86), platelet count (PLT) (*p* = 0.42), creatinine (*p* = 0.074), BUN (*p* = 0.88), or INR (*p* = 0.94). However, hemoglobin (Hgb) levels were significantly lower in the mixed growth group (*p* < 0.001). Regarding operative details, most patients underwent RIRS (90.4% overall), with a slightly higher proportion of URS cases in the mixed growth group (*p* = 0.092). The proportion of pre‐stented ureters was similar (*p* = 0.09), and operative time was comparable between groups (*p* = 0.34).

### 3.2 Correlation Between Variables and Mixed Growth

Females had a higher likelihood of mixed growth (*p* < 0.001), while patients with lower hemoglobin levels were significantly more likely to be in the mixed growth group (*p* < 0.001).

Postoperative complications were significantly higher in patients with mixed bacterial growth compared to those with no growth on urine culture. The incidence of urinary tract infection (UTI) was significantly increased in the mixed growth group (17.8% vs. 2.7%, *p* = 0.003). Similarly, readmission rates were markedly higher among patients with mixed growth (17.8% vs. 0.0%, *p* < 0.001), as were postoperative fever rates (15.1% vs. 0.0%, *p* < 0.001).

Although ICU admission occurred only in the mixed growth group (4.1% vs. 0.0%), this difference did not reach statistical significance (*p* = 0.08) (Table 1).

**Table 1:** Patient characteristics.

	No growth *N* = 73	Mixed growth *N* = 73	Total *N* = 146	*p* value
Gender, *n* (%)				
Female	22 (30.14)	48 (65.75)	70 (47.95)	< 0.001
Male	51 (69.86)	25 (34.25)	76 (52.05)	
BMI	29.0 (7.5)	28.4 (6.0)	28.7 (6.8)	0.62
Ethnicity, *n* (%)				
Non‐Arab	43 (58.9)	38 (52.1)	81 (55.5)	0.41
Arab	30 (41.1)	35 (47.9)	65 (44.5)	
Co‐morbidities, *n* (%)				
DM (No)	55 (75.3)	62 (84.9)	117 (80.1)	0.21
HTN (No)	60 (82.2)	59 (80.8)	119 (81.5)	0.83
Anemia (No)	66 (90.4)	60 (82.2)	126 (86.3)	0.15
Dyslipidemia (No)	70 (95.9)	66 (90.4)	136 (93.2)	0.19
Preoperative laboratory, mean (SD)				
WBC	8.3 (2.4)	8.4 (2.9)	8.3 (2.7)	0.86
Hgb	13.7 (1.7)	12.5 (1.9)	13.1 (1.9)	< 0.001
PLT	314.4 (100.0)	302.4 (76.1)	308.4 (88.7)	0.42
Creatinine	85.2 (31.0)	76.1 (30.2)	80.6 (30.8)	0.074
BUN	4.8 (2.4)	4.7 (2.4)	4.8 (2.4)	0.88
INR Median (IQR)	1.0 (1.0–1.0)	1.0 (1.0–1.0)	1.0 (1.0–1.0)	0.94
CFU/uL in urine Median (IQR)	57.0 (17.0–120.0)	148.0 (82.0–440.0)	95.5 (38.0–275.0)	< 0.001
Type of surgery				
URS	4 (5.5)	10 (13.7)	14 (9.6)	0.092
RIRS	69 (94.5)	63 (86.3)	132 (90.4)	
Pre‐stented ureter (Yes)	63 (86.3)	55 (75.3)	118 (80.8)	0.09
Operative time min, mean (SD)	89.5 (45.6)	82.7 (41.5)	86.1 (43.6)	0.34
Postoperative complications, *n* (%)				
UTI (Yes)	2 (2.7)	13 (17.8)	15 (10.3%)	0.003*
Readmission (Yes)	0 (0.0)	13 (17.8)	13 (8.9)	< 0.001*
Fever (Yes)	0 (0.0)	11 (15.1)	11 (7.5)	< 0.001*
ICU admission (Yes)	0 (0.0)	3 (4.1)	3 (2.1)	0.08

*Note:* Continuous variables are presented as mean (standard deviation) or median (interquartile range), as appropriate, and categorical variables are expressed as number (percentage). Statistical comparisons were performed using the chi‐square test or Fisher's exact test for categorical variables, and the independent *t*‐test or Mann–Whitney *U* test for continuous variables, as appropriate, *p < 0.05.

Abbreviations: BMI, body mass index; BUN, blood urea nitrogen; CFU, colony‐forming units; DM, diabetes mellitus; Hgb, hemoglobin; HTN, hypertension; ICU, intensive care unit; INR, international normalized ratio; PLT, platelet count; URS, ureteroscopy; RIRS, retrograde intrarenal surgery; UTI, urinary tract infection; WBC, white blood cell count.

In the univariate analysis, male gender was significantly associated with a lower likelihood of mixed bacterial growth (OR = 0.22, 95% CI: 0.11–0.45, *p* = 0.001), indicating a higher prevalence among females. Additionally, mixed growth was associated with increased odds of postoperative urinary tract infection (UTI); however, this did not reach statistical significance (OR = 7.6, 95% CI: 1.66–35.4, *p* = 0.09).

In the multivariate analysis, after adjusting for potential confounders, male gender remained independently associated with a lower likelihood of mixed bacterial growth (OR = 0.25, 95% CI: 0.11–0.52, *p* = 0.001) (Table 2).

**Table 2:** Univariate and multivariate logistic regression analyses of factors associated with mixed bacterial growth on preoperative urine culture.

Variables	Univariate			Multivariate		
	OR	*p* value	95% CI	OR	*p* value	95% CI
Gender (male)	0.22	0.001*	0.11–0.45	0.25	0.001*	0.11–0.52
Stone number	0.99	0.97	0.70–1.4	1.25	0.32	0.79–1.96
UTI (yes)	7.6	0.09	1.66–35.4	15.0	0.003*	2.57–87.86
Stone burden (mm)	0.95	0.02	0.91–0.99	1	—	—

*Note:* Odds ratios (ORs) with 95% confidence intervals (CIs) are reported. Variables with clinical relevance or those demonstrating a *p*‐value < 0.10 in univariate analysis were included in the multivariate model. *p‐value < 0.05 was considered statistically significant.

Abbreviations: CI, confidence interval; OR, odds ratio; UTI, urinary tract infection.

## 4 Discussion

Preoperative urine cultures (UCs) are widely used as a screening tool to identify patients at risk of urinary tract infections (UTIs) and to prevent complications during or after surgical procedures [16, 22]. However, contamination of UCs can lead to false‐positive or mixed bacterial growth, potentially resulting in inappropriate antibiotic use [18, 19, 23, 24]. In the absence of additional patient‐related risk factors [25]. To our knowledge, few studies have evaluated risk factors for mixed‐growth preoperative UCs and their association with postoperative complications following ureteroscopy with laser lithotripsy.

This study included 146 patients, divided equally into two groups: 73 with no bacterial growth on urine culture and 73 with mixed growth. The cohort comprised 52.05% males and 47.95% females, with a statistically significant difference in gender distribution (*p* < 0.001), as females were more likely to have mixed growth.

Female sex was identified as a significant risk factor for mixed‐growth urine cultures. These findings are consistent with previous studies demonstrating that female sex and increased BMI are independent risk factors for urine culture contamination [26, 27, 28]. In terms of BMI, prior studies identified it as a risk factor [26, 27, 28], this was not observed in our cohort. This association may be attributed to anatomical and physiological differences, including external genital anatomy and body habitus, which can complicate proper urine collection [26, 29]. Conversely, patients with a preoperative ureteral stent demonstrated lower odds of contamination compared to those without a stent, possibly reflecting differences in urinary drainage or clinical management, although patients with indwelling stents are generally at higher risk of bacteriuria and stent‐related symptoms [30].

Regression analysis further supported these findings. In the univariate analysis, male gender was significantly associated with a lower likelihood of mixed growth cultures (OR = 0.22, 95% CI: 0.11–0.45, *p* = 0.001), indicating that females had a higher probability of contamination. This is consistent with prior studies identifying female gender as a risk factor for both contaminated urine cultures and postoperative UTIs [31, 32, 33], likely due to anatomical factors such as a shorter urethra and proximity to the perineal region.

A statistically significant association was observed between mixed bacterial growth and postoperative UTI following ureteroscopy (*p* = 0.003). Importantly, patients with mixed growth on preoperative urine cultures exhibited a higher risk of postoperative UTI compared to those with no growth. This finding contrasts with the results of Polin et al., who reported no significant difference in postoperative UTI rates between patients with mixed flora and those with negative urine cultures (13% vs. 14%, *p* = 0.77) [34], Additionally, some studies have suggested that mixed growth may be considered equivalent to negative cultures, without an increased risk of postoperative complications, supporting the avoidance of unnecessary preoperative antibiotics in patients undergoing uncomplicated ureteroscopy for stone disease [35]. In contrast, our findings demonstrate an increased risk of postoperative complications, including UTIs, among patients with mixed growth, highlighting the need for careful preoperative evaluation and risk stratification in this population.

Our findings underscore the importance of preoperative risk stratification and patient selection. Female patients and those with preoperative UTIs may require more intensive evaluation and monitoring to mitigate the risk of residual stones and complications. Additionally, the lack of association between stone number and surgical outcomes highlights the need to prioritize stone burden when planning interventions.

### 4.1 Limitations

This study has several limitations. Due to its retrospective nature, we could not accurately determine the duration of pre‐procedural ureteral stents in some cases, which has been previously identified as a risk factor for UTI after ureteroscopy [33]. Additionally, the strict inclusion and exclusion criteria limited the total number of patients available for analysis. Sample size is a common limitation in studies evaluating postoperative UTI following endoscopic urological procedures, given the relatively low incidence of this complication. Furthermore, the study population was largely representative of patients at lower risk for postoperative infectious complications. In particular, no patients with struvite stones were included, and the use of postoperative antibiotics may have influenced the observed incidence of UTI. Consequently, our findings may have limited generalizability to higher‐risk populations, including patients with infection‐related stones or other established risk factors for postoperative UTI. These factors should be considered when interpreting and applying our findings.

## 5 Conclusion

Mixed‐growth urine culture was associated with an increased risk of adverse postoperative infectious outcomes, including UTI, fever, and readmission, in patients undergoing RIRS and R‐URS. Female gender was independently associated with a higher likelihood of mixed growth, suggesting potential underlying biological or microbiological differences. These findings underscore the clinical importance of recognizing mixed growth as a potential risk marker rather than a benign finding. Enhanced preoperative evaluation and optimization strategies may help mitigate postoperative complications in this high‐risk group. Further prospective studies are warranted to validate these findings and guide evidence‐based management.

## Author Contributions

**Khalid Elsayed Mahmoud:** conceptualization, methodology, investigation, writing – review and editing, writing – original draft. **Ahmad R. Al‐Qudimat:** conceptualization, validation, methodology, formal analysis, writing – review and editing, writing – original draft. **Ahmed Khaled Alhusban:** methodology, investigation, writing – original draft. **Raed M. Al‐Zoubi:** investigation, writing – original draft. **Mai Elaarag:** investigation, methodology, writing – original draft. **Abdulkader alobiedly:** investigation, writing – original draft, writing – review and editing. **Ibrahim A. khalil:** writing – review and editing. **Abdulla AlAnsari:** conceptualization, supervision, writing – review and editing, writing – original draft.

## Funding

The authors have nothing to report.

## Consent

The requirement for informed consent was waived by the Institutional Review Board owing to the retrospective nature of the study.

## Conflicts of Interest

The authors declare no conflicts of interest.

## AI Use Statement

During the preparation of this manuscript, the authors used artificial intelligence (AI) tools solely to assist with language editing. The authors reviewed and edited the AI‐xassisted content and take full responsibility for the final content of the manuscript.

## Transparency Statement

Ahmad R. Al‐Qudimat affirms that this manuscript is an honest, accurate, and transparent account of the study being reported; that no important aspects of the study have been omitted; and that any discrepancies from the study as planned (and, if relevant, registered) have been explained.

## Data Availability

The data that support the findings of this study are available from the corresponding author upon reasonable request.

## References

[1] C. D. Scales, Jr. , A. C. Smith , J. M. Hanley , and C. S. Saigal , “Prevalence of Kidney Stones in the United States,” European Urology 62, no. 1 (2012): 160–165. 22498635 10.1016/j.eururo.2012.03.052 PMC3362665

[2] E. N. Taylor , “Obesity, Weight Gain, and the Risk of Kidney Stones,” Journal of the American Medical Association 293, no. 4 (2005): 455–462. 15671430 10.1001/jama.293.4.455

[3] H. Shehadeh , W. El Ansari , M. Alkabbani , et al., “Understanding the Influence of Demographic and Clinical Factors on Urinary Stone Composition: A Retrospective Multivariate Study in a Diverse Population,” UroPrecision 3, no. 3 (2025): 161–168.

[4] C. Türk , A. Petřík , K. Sarica , et al., “EAU Guidelines on Diagnosis and Conservative Management of Urolithiasis,” European Urology 69, no. 3 (2016): 468–474. 26318710 10.1016/j.eururo.2015.07.040

[5] R. M. Geraghty , P. Jones , and B. K. Somani , “Worldwide Trends of Urinary Stone Disease Treatment Over the Last Two Decades: A Systematic Review,” Journal of Endourology 31, no. 6 (2017): 547–556. 28095709 10.1089/end.2016.0895

[6] J. de la Rosette , J. Denstedt , P. Geavlete , et al., “The Clinical Research Office of the Endourological Society Ureteroscopy Global Study: Indications, Complications, and Outcomes in 11,885 Patients,” Journal of Endourology 28, no. 2 (2014): 131–139. 24147820 10.1089/end.2013.0436

[7] D. Assimos , A. Krambeck , N. L. Miller , et al., “Surgical Management of Stones: American Urological Association/Endourological Society Guideline, Part I,” Journal of Urology 196, no. 4 (2016): 1153–1160. 27238616 10.1016/j.juro.2016.05.090

[8] M. C. Lee and S. V. Bariol , “Evolution of Stone Management in Australia,” supplement, BJU International 108, no. Suppl 2 (2011): 29–33. 22085123 10.1111/j.1464-410X.2011.10695.x

[9] M. Ordon , D. Urbach , M. Mamdani , R. Saskin , R. J. D'A Honey , and K. T. Pace , “The Surgical Management of Kidney Stone Disease: A Population Based Time Series Analysis,” Journal of Urology 192, no. 5 (2014): 1450–1456. 24866599 10.1016/j.juro.2014.05.095

[10] G. M. Preminger , H. G. Tiselius , D. G. Assimos , et al., “2007 Guideline for the Management of Ureteral Calculi,” Journal of Urology 178, no. 6 (2007): 2418–2434. 17993340 10.1016/j.juro.2007.09.107

[11] P. Geavlete , D. Georgescu , G. Niţă , V. Mirciulescu , and V. Cauni , “Complications of 2735 Retrograde Semirigid Ureteroscopy Procedures: A Single‐Center Experience,” Journal of Endourology 20, no. 3 (2006): 179–185. 16548724 10.1089/end.2006.20.179

[12] E. Perez Castro , P. J. S. Osther , V. Jinga , et al., “Differences in Ureteroscopic Stone Treatment and Outcomes for Distal, Mid‐, Proximal, or Multiple Ureteral Locations: The Clinical Research Office of the Endourological Society Ureteroscopy Global Study,” European Urology 66, no. 1 (2014): 102–109. 24507782 10.1016/j.eururo.2014.01.011

[13] M. Grabe , “Controversies in Antibiotic Prophylaxis in Urology,” supplement, International Journal of Antimicrobial Agents 23, no. Suppl 1 (2004): 17–23. 15037324 10.1016/j.ijantimicag.2003.09.005

[14] B. K. Somani , G. Giusti , Y. Sun , et al., “Complications Associated With Ureterorenoscopy (URS) Related to Treatment of Urolithiasis: The Clinical Research Office of Endourological Society URS Global Study,” World Journal of Urology 35, no. 4 (2017): 675–681. 27492012 10.1007/s00345-016-1909-0 PMC5364249

[15] A. N. Norsworthy and M. M. Pearson , “From Catheter to Kidney Stone: The Uropathogenic Lifestyle of *Proteus mirabilis* ,” Trends in Microbiology 25, no. 4 (2017): 304–315. 28017513 10.1016/j.tim.2016.11.015 PMC5365347

[16] D. J. Lightner , K. Wymer , J. Sanchez , and L. Kavoussi , “Best Practice Statement on Urologic Procedures and Antimicrobial Prophylaxis,” Journal of Urology 203, no. 2 (2020): 351–356. 31441676 10.1097/JU.0000000000000509

[17] P. Motamedinia , R. Korets , G. Badalato , and M. Gupta , “Perioperative Cultures and the Role of Antibiotics During Stone Surgery,” Translational Andrology and Urology 3, no. 3 (2014): 297–301. 26816781 10.3978/j.issn.2223-4683.2014.07.01 PMC4708583

[18] M. T. LaRocco , J. Franek , E. K. Leibach , et al., “Effectiveness of Preanalytic Practices on Contamination and Diagnostic Accuracy of Urine Cultures: A Laboratory Medicine Best Practices Systematic Review and Meta‐Analysis,” Clinical Microbiology Reviews 29, no. 1 (2016): 105–147. 26598386 10.1128/CMR.00030-15 PMC4771218

[19] E. M. Burd and K. S. Kehl , “A Critical Appraisal of the Role of the Clinical Microbiology Laboratory in the Diagnosis of Urinary Tract Infections,” supplement, Journal of Clinical Microbiology 49, no. 9 (September 2011): S34–S38, 10.1128/JCM.00788-11.

[20] N. Karah , R. Rafei , W. Elamin , et al., “Guideline for Urine Culture and Biochemical Identification of Bacterial Urinary Pathogens in Low‐Resource Settings,” Diagnostics 10, no. 10 (2020): 832. 33081114 10.3390/diagnostics10100832 PMC7602787

[21] A. Skolarikos , R. Geraghty , B. Somani , et al., “European Association of Urology Guidelines on the Diagnosis and Treatment of Urolithiasis,” European Urology 88, no. 1 (2025): 64–75. 40268592 10.1016/j.eururo.2025.03.011

[22] G. Bonkat , T. Cai , C. Galeone , B. Koves , and F. Bruyere , “Adherence to European Association of Urology Guidelines and State of the Art of Glycosaminoglycan Therapy for the Management of Urinary Tract Infections: A Narrative Review and Expert Meeting Report,” European Urology Open Science 44 (2022): 37–45. 36051173 10.1016/j.euros.2022.07.009 PMC9424561

[23] M. E. Lough , E. Shradar , C. Hsieh , and H. Hedlin , “Contamination in Adult Midstream Clean‐Catch Urine Cultures in the Emergency Department: A Randomized Controlled Trial,” Journal of Emergency Nursing 45 (2019): 488–501. 31445626 10.1016/j.jen.2019.06.001

[24] R. Eley , C. Judge , L. Knight , G. Dimeski , and M. Sinnott , “Illustrations Reduce Contamination of Midstream Urine Samples in the Emergency Department,” Journal of Clinical Pathology 69 (2016): 921–925. 26893403 10.1136/jclinpath-2015-203504

[25] B. H. Chew , N. L. Miller , J. E. Abbott , et al., “A Randomized Controlled Trial of Preoperative Prophylactic Antibiotics Prior to Percutaneous Nephrolithotomy in a Low Infectious Risk Population: A Report From the EDGE Consortium,” Journal of Urology 200, no. 4 (2018): 801–808. 29684391 10.1016/j.juro.2018.04.062

[26] P. S. Whelan , A. Nelson , C. J. Kim , et al., “Investigating Risk Factors for Urine Culture Contamination in Outpatient Clinics: A New Avenue for Diagnostic Stewardship,” Antimicrobial Stewardship & Healthcare Epidemiology 2, no. 1 (2022): e29. 35445218 10.1017/ash.2021.260 PMC9016366

[27] W. Ito , N. Choi , G. Letner , et al., “Preoperative Urine Culture With Contaminants Is Not Associated With Increased Risk for Urinary Tract Infection After Ureteroscopic Stone Treatment,” World Journal of Urology 42, no. 1 (2024): 159. 38488875 10.1007/s00345-024-04793-w

[28] M. A. Hansen , M. Valentine‐King , R. Zoorob , et al., “Prevalence and Predictors of Urine Culture Contamination in Primary Care: A Cross‐Sectional Study,” International Journal of Nursing Studies 134 (2022): 104325. 35914376 10.1016/j.ijnurstu.2022.104325 PMC10513105

[29] B. O Leary , F. Armstrong , S. Byrne , A. F. Talento , and S. O'Coigligh , “The Prevalence of Positive Urine Dipstick Testing and Urine Culture in the Asymptomatic Pregnant Woman: A Cross‐Sectional Study,” European Journal of Obstetrics & Gynecology and Reproductive Biology 253 (2020): 103–107. 32862029 10.1016/j.ejogrb.2020.08.004

[30] D. Abt , E. Warzinek , H. Schmid , S. Haile , and D. Engeler , “Influence of Patient Education on Morbidity Caused by Ureteral Stents: Patient Education on Ureteral Stents,” International Journal of Urology: Official Journal of the Japanese Urological Association 22 (2015): 679–683. 25882159 10.1111/iju.12782

[31] H. J. Chung , A. T. L. Lin , C. C. Lin , T. J. Chen , and K. K. Chen , “Patients With Urinary Incontinence Appear More Likely to Develop Upper Urinary Tract Stones: A Nationwide, Population‐Based Study With 8‐Year Follow‐Up,” PLoS One 11, no. 8 (2016): e0161223. 27536881 10.1371/journal.pone.0161223 PMC4990176

[32] C. Peng , Z. Chen , and J. Xu , “Risk Factors for Urinary Infection After Retrograde Upper Urinary Lithotripsy: Implication for Nursing,” Medicine 100, no. 31 (2021): e26172. 34397789 10.1097/MD.0000000000026172 PMC8341329

[33] S. Chugh , A. Pietropaolo , E. Montanari , K. Sarica , and B. K. Somani , “Predictors of Urinary Infections and Urosepsis After Ureteroscopy for Stone Disease: A Systematic Review From EAU Section of Urolithiasis (EULIS),” Current Urology Reports 21, no. 4 (2020): 16. 32211969 10.1007/s11934-020-0969-2

[34] M. R. Polin , A. Kawasaki , C. L. Amundsen , A. C. Weidner , and N. Y. Siddiqui , “Do Mixed‐Flora Preoperative Urine Cultures Matter?,” Southern Medical Journal 110 (2017): 426–429. 28575903 10.14423/SMJ.0000000000000659

[35] J. K. Kim , E. J. Margolin , D. L. Barquin , et al., “Infection Risk in Patients With Mixed Flora in Urine Cultures Prior to Ureteroscopy,” Journal of Endourology 39, no. 8 (2025): 808–814. 39925120 10.1089/end.2024.0670

